# The Synergistic Effect of Nanocrystals Combined With Ultrasound in the Generation of Reactive Oxygen Species for Biomedical Applications

**DOI:** 10.3389/fbioe.2019.00374

**Published:** 2019-11-26

**Authors:** Veronica Vighetto, Andrea Ancona, Luisa Racca, Tania Limongi, Adriano Troia, Giancarlo Canavese, Valentina Cauda

**Affiliations:** ^1^Department of Applied Science and Technology, Politecnico di Torino, Turin, Italy; ^2^Ultrasounds and Chemistry Lab, Advanced Metrology for Quality of Life, Istituto Nazionale di Ricerca Metrologica, Turin, Italy

**Keywords:** zinc oxide, nanocrystals, ultrasound, cavitation, reactive oxygen species, contrast agent

## Abstract

Reactive oxygen species (ROS) effects on living cells and tissues is multifaceted and their level or dose can considerably affect cell proliferation and viability. It is therefore necessary understand their role also designing ways able to regulate their amount inside cells, i.e., using engineered nanomaterials with either antioxidant properties or, for cancer therapy applications, capable to induce oxidative stress and cell death, through tunable ROS production. In this paper, we report on the use of single-crystalline zinc oxide (ZnO) round-shaped nanoparticles, yet ZnO nanocrystals (NCs) functionalized with amino-propyl groups (ZnO-NH_2_ NCs), combined with pulsed ultrasound (US). We show the synergistic effects produced by NC-assisted US which are able to produce different amount of ROS, as a result of inertial cavitation under the pulsed US exposure. Using Passive Cavitation Detection (PCD) and Electron Paramagnetic Resonance (EPR) spectroscopy, we systematically study which are the key parameters, monitoring, and influencing the amount of generated ROS measuring their concentration in water media and comparing all the results with pure water batches. We thus propose a ROS generation mechanism based on the selective application of US to the ZnO nanocrystals in water solutions. Ultrasound B-mode imaging is also applied, proving in respect to pure water, the enhanced ecographic signal generation of the aqueous solution containing ZnO-NH_2_ NCs when exposed to pulsed ultrasound. Furthermore, to evaluate the applicability of ZnO-NH_2_ NCs in the biomedical field, the ROS generation is studied by interposing different tissue mimicking materials, like phantoms and *ex vivo* tissues, between the US transducer and the sample well. As a whole, we clearly proof the enhanced capability to produce ROS and to control their amount when using ZnO-NH_2_ NCs in combination with pulsed ultrasound anticipating their applicability in the fields of biology and health care.

## Introduction

Reactive Oxygen Species (ROS) are the result of partial reduction of molecular oxygen (O_2_) (Dabrowski, 2017). The hydroxyl radical (HO·) is one of the strongest radicals ever described (Dabrowski, 2017) and it possesses the highest reduction potential of all the ROS that are physiologically relevant: due to its nature it can react with a large variety of different type of biological molecule (Fu et al., 2014).

Nevertheless, oxygen is the fundamental element needed for the normal metabolic activity of every aerobic organism, and so ROS are inevitably produced inside living organisms, as cells. ROS are normally involved in different cell functions as signaling system, induction of mitogenic response, and mitochondria activity (Fu et al., 2014). Nonetheless, the survival of cells is related to the ability of maintaining the redox homeostasis (Dabrowski, 2017) during all this processes. An instability in this equilibrium results in a variety of possible different diseases. When chronic low levels of ROS occurs in a biological living system indeed, it has been demonstrated that gene mutation and malignant cell transformation can appear, or a large variety of vascular diseases can be promoted (Lau et al., 2008). In addition, Shafique et al. (2013) established that the increase in ROS levels can have a protective role in endothelial homeostasis, improving the vascular function in patients affected by cardio vascular disease (CDV). It is also been proven that the ROS generation achieved by the external activation of membrane-bound NADPH oxidase can induce angiogenesis and other essential functions of endothelial cells, such as hemostasis (Kim et al., 2017; Aldosari et al., 2018). The activation of angiogenesis caused by an increase in ROS production to restore ROS physiological levels, can be beneficial not only for CVD, as occurs after ischemia (Urao et al., 2008), but can also contribute to wound healing (Osumi et al., 2017). On the other hand, an excessive production of ROS leads to a disequilibrium redox state, where the antioxidant defenses of the cell has been overcome, being responsible for damaging cellular components, as lipids, proteins and DNA. Acute high levels of ROS cause the activation of different signal pathways, involving cytokines, transcription factors, and mediators, responsible for cell death, causing ROS-mediated apoptosis or necrosis (Dabrowski, 2017). These effects generated by cellular oxidative stress can be exploited for cancer therapeutic applications (Pelicano et al., 2004; Nogueira and Hay, 2013; Tong et al., 2015).

Therefore, it is clear that the ability to regulate the amount of ROS generated inside cells plays a fundamental role in the survival or death of cells. During last years thus different ways to produce ROS in a controlled manner were investigated. Nanomaterials (NM) are largely studied with the aim to apply them in biomedical field, and one of the principal mechanisms of nanotoxicity is the production of oxidative stress due to ROS generation. It has also been demonstrated that the level of generated ROS is dependent on the physical and chemical properties of the considered engineered nanomaterial, as size, surface to volume ratio, and surface reactivity (Gonzalez et al., 2008; Abdal Dayem et al., 2017). Carlson et al. (2008) measured the amount of ROS produced in cells when Ag nanoparticles with different dimensions were present: 10-fold increase of ROS levels was measured in cells exposed to the smallest dimension nanoparticles. In the same work, it has been assessed that not all nanomaterials with equal dimensions can produce the same amount of ROS, supporting the idea that the ROS generation from NM depends also on their chemical nature.

Oxidative stress can also be achieved by an external activation of NMs to generate ROS, leading to tumor cell death under specific conditions. An example of this mechanism is photodynamic therapy (PDT) (Dabrowski, 2017). We have previously (Ancona et al., 2018) proposed the use of hybrid nanoparticles, able to produce intracellular ROS only when remotely activated by UV light irradiation. The photogeneration of electrons (e^−^) and holes (h^+^) have the ability to react with the environment forming superoxide radical anions (O^2−^) when e^−^ reduce oxygen molecules while hydroxyl radicals (HO·) and hydrogen peroxide (H_2_O_2_) molecules are produced when h^+^ oxidize water molecules. In the last two decades, the use of photosensitizer materials in PDT was largely applied to cancer therapy (Dougherty et al., 1998; Dolmans et al., 2003; Dos Santos et al., 2019), but there are some limitations, as the limited tissue penetration depth of UV light used to excite the photosensitizer, that confine the application of PDT to treat superficial tumors (Dabrowski, 2017).

Ultrasound (US) is another external stimulus investigated to activate the production of ROS and sonodynamic therapy (SDT) is recently emerged as an alternative to PDT due to the higher penetration depth of ultrasound with respect to UV light (McHale et al., 2016). Additionally, under ultrasound excitation, cavitation bubbles are generated and their violent oscillation and collapse let them act as nano-chemical reactors, leading to the formation of ROS in water media. The compounds that promote ROS formation, chemically reacting or introducing a larger amount of bubbles, are named sonosensitizers (Yasuda et al., 2015). Most of the sonosensitizers, such as porphyrins, are characterized by an easy aggregation in physiological environment due to their hydrophobic nature, decreasing the therapy effectiveness, by an intrinsic toxicity and by minor selectivity to cancer tissue (Canavese et al., 2018). The effectiveness of SDT is related to the ability of efficiently generating ROS, without major drawbacks related to the nature of the implied sonosensitizer material.

A similar and most conventional technique, using photosensitizers to produce ROS is the PDT. In this treatment UV light at a specific wavelength excites the photosensitizer molecules to obtain different species of ROS and subsequent cancer cell death. PDT has been employed with promising results for the treatment of bladder, esophagus, skin, and others cancers, and is at the stage of clinical evaluation (van Straten et al., 2017). A possibility, is to combine the use of ZnO with UV in a novel PDT approach: ZnO nanoparticles have been actually employed as carrier of a photosensitizer and other chemotherapeutics (Zhang et al., 2011; Firdous, 2018) or directly as photosensitizer, as we reported recently (Ancona et al., 2018), generating superoxide, hydrogen peroxide, and hydroxyl radicals and decreasing HeLa cells viability upon irradiation. However, the main limitation of PDT is the poor tissue penetration of light, in particular the UV, that limits PDT for the treatment of superficial tumors, as melanomas. In order to overcome this drawback, a possible solution could be “tune” ZnO absorption near visible light (with increased tissue penetration rate), enveloping ZnO nanoparticles into other metals or doping them in various manners (Hu et al., 2013).

In this study, Zinc Oxide nanocrystals with a functionalized surface of aminopropyl groups (ZnO-NH_2_ NCs) have been proved able to produce ROS in a controlled manner, when stimulated by US generated by an already approved medical device (LipoZero G39).

Nanosized ZnO is a metal oxide well-known for its safety in biomedical fields (Racca et al., 2018). In this work, we demonstrated that our customized ZnO-NH_2_ NCs specific monocrystalline structure, size, shape, and functionalization, are able to generate a tunable quantity of ROS according to the intensity od administered US. More in details, the ultrasound is generated through the use of a safe medical device able to generate cavitation phenomena in human tissues. Several parameters like US output power, frequency, duty cycle, sonication time, as well as ZnO-NH_2_ NCs concentration in water media, were systematically examined. To push our study forward up to a possible *in vivo* application, it has also been verified that a larger amount of controllably cavitation and ROS generation occur also when tissue mimicking materials have been employed.

All the presented results are thus preliminary data which can potentially bring to the safe and reproducible use of nanocrystals-assisted ultrasounds for *in vivo* application, going from either tissue engineering proliferative effects to anticancer therapies application, thanks to the high control achieved on the amount of generated ROS.

## Materials and Methods

### ZnO-NH_2_ NCs Synthesis and Functionalization

ZnO nanoparticles were synthesized through a microwave-assisted synthesis, as previously reported (Garino et al., 2019a). The reaction path is based on the hydrolysis of the zinc precursors (zinc acetate dihydrate) due to the presence of sodium hydroxide as the base in methanol. The as-synthesized ZnO were then functionalized with amino-propyl groups with a post-grafting approach using 3-(AminoPropyl)-TriEthoxySilane (APTES) at 10 mol% with respect to the molar amount of ZnO, as in Dumontel et al. (2017) and Garino et al. (2019a).

The obtained nanostructures are amine-functionalized zinc oxide nanocrystals (ZnO-NH_2_ NCs) stable colloidal suspensions in ethanol.

### ZnO-NH_2_ NCs Characterization

The morphological characterization of ZnO-NH_2_ NCs was performed by both Field Emission Scanning Electron Microscopy (FESEM, Carl Zeiss Merlin) and Transmission Electron Microscopy (TEM, FEI Tecnai operating at 200 kV) by spotting a diluted ethanolic solution of the samples (100 μg/ml) on a silicon wafer for FESEM or on copper grid with 300 carbon mesh for TEM, respectively. The particles size and Z-potential value of ZnO-NH_2_ NCs in water suspension was determined by the Dynamic Light Scattering (DLS) technique (Zetasizer Nano ZS90, Malvern).

The crystalline structure of ZnO-NH_2_ NCs was analyzed by X-Ray Diffraction (XRD) with a Panalytical X'Pert diffractometer in Bragg Brentano configuration (Cu-Kα radiation, λ = 1.54 Å, 40 kV, and 30 mA).

### Evaluation of ROS Production

Ultrasound excitation was carried out with LipoZero G39 (GLOBUS) and the evaluation of ROS production was provided by Electron Paramagnetic Resonance (EPR) Spectroscopy (EMXNano X-Band spectrometer from Bruker) assisted by a spin-trapping technique. The formation of hydroxyl and superoxide anion radicals was actually detected in double distilled water using as a spin trap the 5,5-dimethyl-L-pyrroline-N-oxide (DMPO, Sigma) and each tested sample contained DMPO 10 mM. This compound is suitable for the study of ROS generation due to its capability to trap both hydroxy and superoxide anion radicals. After the ultrasound irradiation, the sample was promptly transferred into a quartz microcapillary tube and inserted in the EPR cavity. The spectra were recorded with the following measurement conditions: center field 3428 G, sweep time 160.0 s, sample g-factor 2.00000, number of scans 15. After acquisition, the spectrum was processed using the Bruker Xenon software (Bruker) for baseline correction. Analysis of recorded spectra was executed using the Bruker SpinFit software.

To perform sonication, 1 ml of sample was placed in a 24 well plate (Thermo Scientific) which was positioned in contact with LipoZero transducer through a thin layer of coupling gel (Stosswellen Gel Bestelle, ELvation Medical GmbH). Formation of hydroxyl and superoxide anion radicals was evaluated under a large range of different conditions. Samples were tested for three different sonication times (2, 5, and 10 min), five Duty Cycle conditions (10, 20, 30, 40, and 50%), three distinct working frequencies (150 KHz, 526 KHz, 1 MHz) and different output powers of the LipoZero device (0.3, 0.6, 0.9, 1.2, and 1.5 W/cm^2^ corresponding to 10, 20, 30, 40, and 50% of the maximum output power). In addition to these conditions, different concentrations of amino-functionalized ZnO-NH_2_ NCs were also tested (50, 100, and 200 μg/ml) for oxygen radicals production. Temperature inside the sample well was monitored by a temperature Multilogger Thermometer 502A1 (TERSID S.r.l.).

### Needle Cavitometer Measurements

The acoustic pressure reached inside the well with LipoZero G39 at different output powers (0.6, 0.9, 1.2, and 1.5 W/cm^2^) and the acoustic cavitation activity in presence or absence of nanocrystals was monitored by recording the broad band acoustic emissions generated by collapsing bubbles by using a needle hydrophone Dapco NP 10-3 coupled to a spectrum analyzer (Agilent N9320B) and integrating the FFT area for a frequency range of 0.8–5.0 MHz. Analyses were performed at least on three spectra for each experiment.

### B-Mode Ecographic Imaging

Ultrasound imaging was performed with a research ultrasonic scanner (Ultrasonix Sonic Touch) equipped with linear probe (L14-5/38) operating at 10 MHz in high resolution mode. It was coupled with the sample holder using ultrasound coupling gel and positioned along the axis of a single plastic well-filled with 1 ml of solution. The imaging transducer was focalized to the excitation transducer focus. Real-time videos of the system response to ultrasound irradiation were recorded and videos were analyzed using MATLAB script which calculated the relative average intensity of the bright spots in the region of interest (ROI) of each frame of the videos. Three videos were recorded for each sample.

### Tissue-Mimicking and Ultrasound Irradiation

In order to evaluate the attenuation of ultrasound effects in the presence of tissue mimicking media, different materials, as phantom and *ex vivo* chicken-breast tissue, were interposed between the transducer and the sample.

**Figure 1:**
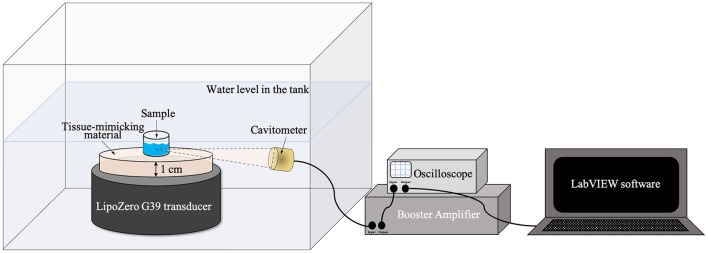
Schematic illustration of ultrasound irradiation set up for the measurements with tissue-mimicking materials.

To conduct these tests, the ultrasound source was immersed in a plexiglass tank filled up with demineralized water and a single well, previously cut and polished from a 24 well plate (Thermo Scientific), was placed at a distance of 1 cm from the transducer surface and exposed to ultrasound, as shown in Figure 1.

Therefore, the measurements were performed using a tissue-mimicking homogeneous phantom (based on 3% in weight of agarose and 0.4 M zinc acetate, Zn(CH_3_COOH)_2_, with an ultrasound attenuation of 0.5 dB/cm·MHz, which matches the attenuation of muscle tissue as reported in Troia et al. (2017) with a diameter of 40 mm and an *ex vivo* tissue (chicken breast); both materials were characterized by a thickness of 1 cm. As a reference, the effects of ultrasound irradiation on the sample were also evaluated considering only the water bath, where the system is immersed, as medium between the piezoelectric transducer and the sample well.

Ultrasound excitation was provided by LipoZero and measurements were conducted with a frequency of 1 MHz, 50% of Duty Cycle, and a power of 3 W/cm^2^ for 20 min. During each experiment the acoustic signal generated inside the well was recorded using a focused piezo-detector (Precision Acoustic) as a cavitometer, coupled to the Booster Amplifier (Precision Acoustic) and connected to a digital oscilloscope (TDS 2012B, Tektronix). To store the data, LabVIEW software was used and 100 μs were recorded every 2 s if the signal measured by the oscilloscope was higher than 0.001 V. Data were successively analyzed with MATLAB software. The time-domain signal was transformed in the frequency-domain by Fourier Transform and the cavitation dose was quantified with MATLAB by calculating the area subtended by the curve. The area measurements considered only values from a frequency of 2.5–12 MHz, in order to eliminate the initial 1 MHz driving signal.

At the end of sonication, EPR spectroscopy assisted by a spin-trapping technique was performed as previously described. In this set of tests, EPR measurement conditions were as follows: center field 3428 G, sweep time 60.0 s, sample g-factor 2.00000, number of scans 15.

To evaluate the increase of ROS production in presence of ZnO-NH_2_, two conditions were tested for each tissue-mimicking material and, as a reference, for water: milliQ water with 20 mM content of DMPO and milliQ water with 20 mM content of DMPO and a concentration of NCs equal to 200 μg/ml.

SigmaPlot 14.0 software was used for all statistical analyses. Data are expressed as the mean ± standard error mean (S.E.M.). Asterisks denoting *P*-values (^*^p < 0.05 and ^**^p < 0.001) and sample sizes are indicated in each figure legend.

## Discussion

**Figure 2:**
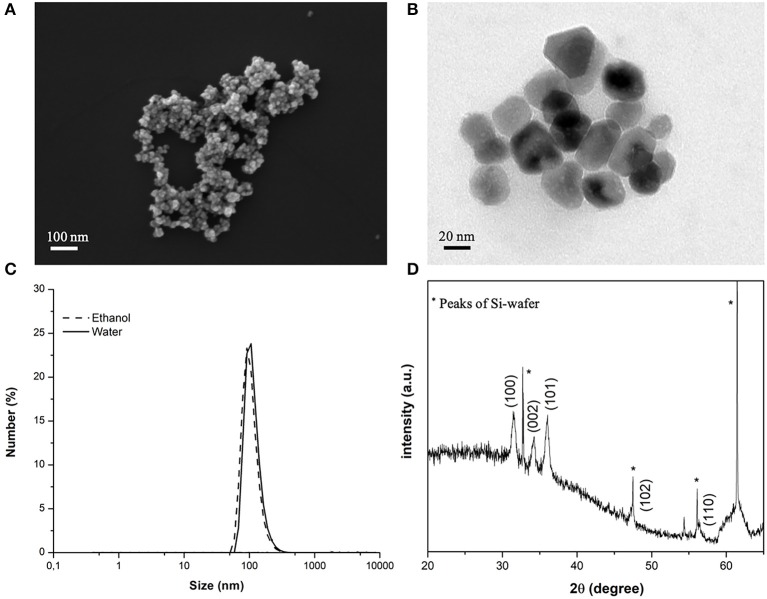
**(A)** FESEM and **(B)** TEM images of the ZnO-NH_2_ NCs used in this study; **(C)** average hydrodynamic diameter in ethanolic solution by DLS and **(D)** XRD pattern of the NCs.

As evidenced by FESEM and TEM analysis (Figures 2A,B), the ZnO-NH_2_ nanomaterials can be ascribed to single nanocrystalline structures, with an average diameter of 20 ± 5 nm, see also Garino et al. (2019a) for comparison. Amine-functionalized ZnO nanocrystals have an average hydrodynamic diameter of 122 nm in their original ethanolic suspension and also in water (Figure 2C) and a Z-Potential value of +22 mV in double distilled water. The XRD pattern in Figure 2D shows the typical hexagonal wurtzitic crystalline structure of zinc oxide materials, confirming also what previously reported so far (Garino et al., 2019a,b).

**Figure 3:**
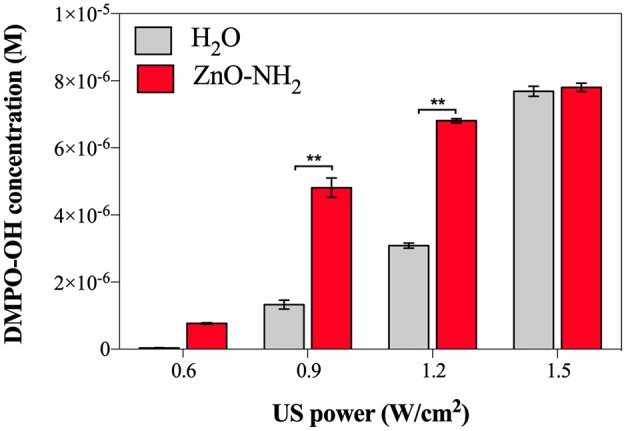
DMPO-OH concentration (M) to evaluate ROS production after 10 min of US irradiation according to different US powers, in presence of ZnO-NH_2_ NCs (200 μg/ml). All measurements were conducted in triplicate with 10% DC, 1 MHz frequency, using the LipoZero transducer. 1-way ANOVA was performed to determine statistical significance (*p < 0.05 and **p < 0.001).

The EPR spectroscopy was used to evaluate the enhancement of ROS production when ZnO-NH_2_ NCs are present in water (with a concentration of 200 μg/ml) and according to different US power. The results are shown in Figure 3. Samples were irradiated with US for 10 min, at a frequency of 1 MHz and the ultrasound stimulation was pulsed, with a Duty Cycle of 10%, Pulse Repetition Frequency 1 Hz. The concentration of DMPO-OH was then evaluated since it is directly correlated to the ROS production, in particular to the hydroxyl and superoxide anions production.

The measured acoustic pressures reached inside the well at the various output powers (0.6, 0.9, 1.2, and 1.5 W/cm^2^) were between 1 and 1.5 MPa, justifying the occurrence of the inertial cavitation inside the sample well, justifying the occurrence of the inertial cavitation inside the sample well. Actually, when a lower output power was used, i.e., 0.3 W/cm^2^ (corresponding to 10% of the maximum output US power), also the threshold for ROS generation was not reached, meaning that inertial cavitation did not occur in the sample during the US irradiation. Otherwise, with 0.6 W/cm^2^ (corresponding to the 20% of the maximum output power), a small amount of hydroxyl and superoxide anions were detected, and even if the amount of DMPO-OH was greater in presence of ZnO-NH_2_ NCs (red bar) with respect to pure water (black bar), it can be noted that the 20% of power was not enough to obtain a statistical difference between samples. A different scenario is depicted when 0.9 and 1.2 W/cm^2^ were utilized. In both cases a significant difference (both with *p* < 0.001) between the amount of ROS produced in pure water and the one obtained in the presence of ZnO-NH_2_ NCs is clearly observed. The results achieved with these conditions indicate the efficacy of our ZnO-NH_2_ NCs to act as an ultrasound responding nano-agent. It is interesting to observe that the power doses of 0.9 and 1.2 W/cm^2^, corresponding to the 30 and 40% of the maximum power output of the Lipozero Transducer, were too low to generate high amount of ROS when the sonicated sample in the well was the pure water. Strikingly, both these low intensities ultrasound conditions are enough to elicit an activation of the ZnO-NH_2_ NCs, widely increasing the amount of ROS produced.

The last US power tested for the sonication was 1.5 W/cm^2^ (corresponding to the 50% of the maximum output power): the delivered intensity of US was sufficiently high to activate the inertial cavitation in the water alone, leading to a large amount of ROS produced, comparable with the one obtained in presence of ZnO-NH_2_ NCs.

From the results in Figure 3, it is assessed that 0.9 W/cm^2^ (30% of US power) is the optimal condition to have ZnO-NH_2_ NCs working as ultrasound responsive nano-agent: the significant difference (*p* < 0.001) in ROS production suggests that, at that power, the US irradiation was not enough intense to cause a large production of hydroxyl and superoxide anions in the water, but it was sufficiently high to initiate the acoustic cavitation in the sample containing ZnO-NH_2_ NCs due to the presence of nanobubbles the NCs surface, which act as nuclei for inertial cavitation and consequently leads to a larger ROS production.

**Figure 4:**
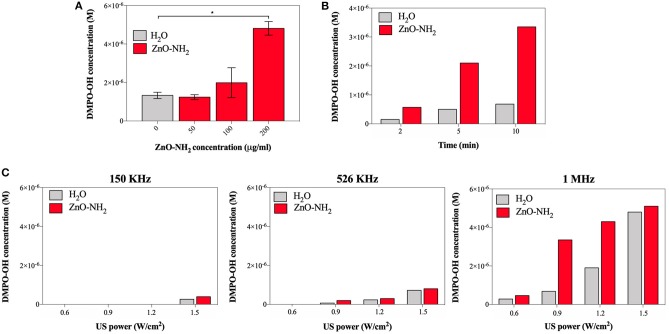
**(A)** Effects on ROS production at different concentration of ZnO-NH_2_ NC; measurements were conducted in duplicate with 10% DC, 0.9 W/cm^2^, 10 min of treatment time, 1 MHz. One-way ANOVA was performed to determine statistical significance (*p < 0.05 and **p < 0.001). **(B)** Evaluation of the optimal amount of treatment time, with 10% DC, 0.9 W/cm^2^, 1 MHz, and 200 μg/ml of ZnO NC. **(C)** Comparison between different excitation frequency for all the tested US power, with 10% of DC, 10 min as treatment and time 200 μg/ml of ZnO NC.

To support these results, other parameters such as ZnO-NH_2_ NCs concentration in water, time of US treatment, frequency of the US were tested to ensure the efficacy of the ZnO-NH_2_ NCs, as reported in Figure 4.

To assess the optimal concentration of ZnO-NH_2_ NCs, 50, 100, and 200 μg/ml of NCs were examined (Figure 4A), suggesting that the highest concentration tested, as used in all the other experiments, is the optimal one. Three different treatment times were thus evaluated, 2, 5, and 10 min, keeping fixed all the other parameters (0.9 W/cm^2^ of power, 10% of DC, and 1 MHz of excitation frequency and 200 μg/ml of ZnO-NH_2_ NCs in water). The insonation time of 10 min was confirmed to be the best treatment time condition to enhance the ultrasound responsive nano-agent capabilities of ZnO-NH_2_ NCs with respect to pure water. In Figure 4C, three excitation frequency were also screened: 150 KHz, 526 KHz, and 1 MHz at different US output powers, with 10% DC and 200 μg/ml as NCs concentration. With the lowest frequency, a large amount of power (50% with respect to the maximum output) was needed to obtain a detectable signal with EPR instrument and DMPO spin adducts. The frequency of 1 MHz, which is the most used frequency for biomedical applications, is here confirmed to enhance the production of ROS in the presence of the ZnO-NH_2_ NC under ultrasound excitation.

An hypothesis for the explanation of ROS generation capabilities of ZnO-NH_2_ NCs is related to the NC surface: the high surface-to-volume ratio of ZnO-NH_2_ NCs, showing a large surface area of 60 m^2^/g [as measured by Nitrogen Sorption isotherm elsewhere (Lops et al., 2019)] and the surface functionalization of amino-propyl groups are both capable to immobilize and promote the inertial cavitation of tiny gas nanobubbles under such US power conditions. As inertial cavitation is produced, ROS are generated: the EPR technique detected OH· radicals, which are one of the most reactive and potentially dangerous species of ROS.

**Figure 5:**
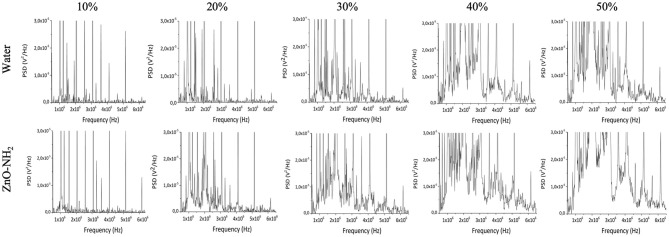
Frequency spectra recorded by needle cavitometer in order to evaluate cavitation of water and ZnO-NH_2_ NCs water suspensions. The comparison between the two solutions was carried out at 1 MHz, 100% of DC, 10 min of insonation with 0.3, 0.6, 0.9, 1.2, and 1.5 W/cm^2^, which correspond to 10, 20, 30, 40, and 50% of maximum available US power.

To evaluate the role of acoustic cavitation on the generation of ROS by the ultrasound exposure of ZnO-NH_2_ NCs passive cavitation detection (PCD) technique was used. Figure 5 shows the frequency spectra of the acoustic signals obtained at different ultrasound intensities with and without ZnO-NH_2_ NCs in solution. At low ultrasound intensities, only harmonics and sub-harmonics signals are present: since these signals are recorded for both water and NCs samples, they are probably due to oscillation of large gas bubbles trapped in the plastic wells of the sample holder. At increasing ultrasound intensities (above 1.2 W/cm^2^), acoustic broadband noise typical of inertial cavitation was recorded for the water solution. When ZnO-NH_2_ NCs were added to the solution, broadband noise signal was recorded at lower ultrasound intensities, suggesting that our NCs acted as nucleation site inducing inertial cavitation, thus decreasing the cavitation threshold. Since it has been shown both theoretically and experimentally that collapsing cavitating bubbles can generate sufficiently high temperatures and pressures able to induce generation of ROS in aqueous solution (The Acoustic Bubble, Leighton), PCD and EPR experiments together suggest that ZnO-NH_2_ NCs generate ROS by inducing inertial cavitation upon ultrasound exposure.

**Figure 6:**
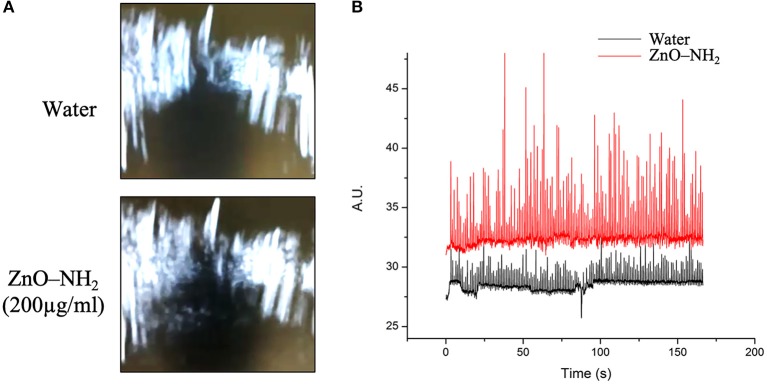
**(A)** B-mode ecographic imaging of the corresponding **(B)** scattering signal related to bubble cavitation events obtained when 50% of maximum available US power was applied at 10% of DC, 1 MHz, and 170 s.

In order to further study the generation of inertial cavitation by ZnO-NH_2_ NCs, ultrasound B-mode imaging was used. Figure 6A shows the ecographic images obtained for water and ZnO-NH_2_ NCs containing solutions exposed to 40% intensity ultrasound. Cavitating bubble generated by ZnO-NH_2_ NCs led to bright spots in the solution, while in the absence of NCs the ecographic signal did not increase. Figure 6B shows the quantification of ecographic contrast obtained during the pulsed ultrasound exposure (170 s), as previously described in the Material and Methods section. ZnO-NH_2_ NCs generated higher ecographic contrast over all the sonication period compared with the water containing solution. Together these results further confirm the ability of ZnO-NH_2_ NCs in inducing inertial cavitation under pulsed ultrasound exposure.

ROS exert a multitude of biological effects (Lau et al., 2008; Racca et al., 2018), which also comprehend the creation of molecular damages inside cells, leading to antitumoral application (de Sá Junior et al., 2017).

In order to evaluate the future applicability of ZnO-NH_2_ NCs in the biomedical field, in particular to subcutaneous *in vivo* applications, the generation of ROS, and the effects of our NCs as ultrasound responsive nano-agent was tested in Phosphate Buffered Saline (PBS) solution, cell culture medium [Minimum Essential Medium Eagle (SIGMA) completed with 10% of Fetal Bovine Serum (FBS, SIGMA) and 1% of Penicillin-Streptomycin] and finally when different tissue mimicking materials were interposed during the insonation between the LipoZero US transducer and the sample well.

The ROS evaluation in PBS and cell culture media are reported in the Supporting Information and confirm the ability of our ZnO-NH_2_ nanocrystals to enhance inertial cavitation and consequently ROS production also in biological media, thus leading to applications *in vitro*.

**Figure 7:**
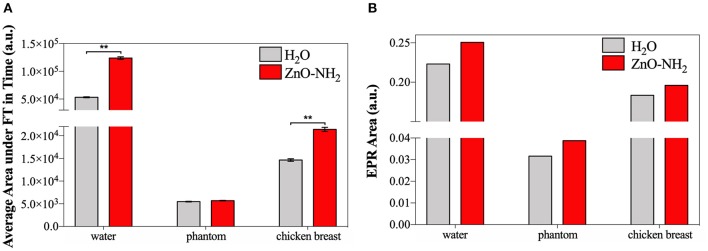
**(A)** Average area under Fourier Transform, calculated with MATLAB, of signals recorded in time by cavitometer during ultrasound irradiation. One-way ANOVA was performed to determine statistical significance (*p < 0.05 and **p < 0.001). **(B)** Area under the EPR spectrum curve measured with Bruker SpinFit software.

The evaluation of the cavitation and ROS generation related to the interposition of tissue-mimicking materials between the ultrasound source and the samples are shown in Figure 7. Different materials were tested, and for all of them the amount of cavitation of water and water with the synergistic effect of ZnO-NH_2_ NCs (200 μg/ml) were evaluated. All the measurements were performed for 20 min, with a frequency of 1 MHz, 50% of Duty Cycle, and 100% of US power available from LipoZero transducer.

Figure 7A exhibits the results of cavitometer measurements during 20 min of insonation, which are the average area under the Fourier Transform (FT) of each measured signal over time. These data correlate with the amount of occurred cavitation, and the S.E.M. is reported. It can be appreciated that, when the ultrasound propagation medium was water, the amount of cavitation detected by the cavitometer was higher than the one measured when tissue mimicking materials were interposed. This effect can be explained considering the attenuation of power perceived inside the sample well. When the phantom and the *ex vivo* tissue are used, the real amount of US energy inside the well is lower, and the measured broadband noise, which correlates to cavitation, reflects this reduction. Nevertheless, a significant increase of cavitation is noticeable when ZnO-NH_2_ NCs are present, not only when water is the transmission medium, but also when *ex vivo* tissue is interposed as the propagation medium. Even if there is not a significant difference in the presence of phantom, an increase of 4% in the generated cavitation can still be noticed when ZnO-NH_2_ NCs are used.

At the end of 20 min of insonation, the amount of hydroxyl and superoxide anion radicals produced were evaluated for all the different conditions using a spin-trapping technique involving DMPO.

Figure 7B shows areas under the EPR spectrum curve, corresponding to the integrated intensity of the radical species and reflecting the concentration of DMPO-OH, index of ROS generation. As similarly reported in Figure 7A, the results in Figure 7B demonstrate that a larger amount ROS were produced when the irradiation medium is distilled water, with respect to phantom and *ex vivo* tissue, The results obtained lead to the conclusion that we successfully generated ROS in a controlled manner even in presence of two different tissue-mimicking materials. The data open the possibility to apply this technology *in vivo* for subcutaneous ROS generation using an already approved medical device.

Despite the general difference between the attenuating media, in all the cases a largest amount of ROS was detected in presence of ZnO-NH_2_ NCs, suggesting that our nanocrystals enhance the production of free radicals under ultrasound stimulation. This phenomenon is proved here also when tissue mimicking materials were interposed between the stimulation source and the sample, suggesting the possibility of *in vivo* applications.

## Conclusion

We report in this paper the ability of ZnO-NH_2_ nanocrystals in inducing inertial cavitation under pulsed ultrasound exposure. In details, it is assessed that 0.9 W/cm^2^ (30% of US power) is the optimal condition to have ZnO-NH_2_ NCs working as ultrasound responsive nano-agent and showing the significant large production of ROS, specifically of hydroxyl and superoxide anions in the water. We proposed, as mechanism of ROS generation, that this US conditions are sufficient to initiate the acoustic cavitation of tiny gas nanobubbles trapped at the ZnO-NH_2_ NCs surface. This inertial cavitation consequently leads to a large ROS production. Strikingly in the same insonating condition, lower cavitation and consequently largely lower amount of ROS are generated from the pure water control sample.

Ultrasound B-mode imaging was also used to confirm the generation of inertial cavitation by ZnO-NH_2_ NCs. An enhanced ecographic signal generation was detected when ZnO-NH_2_ NCs solutions were exposed to 40% intensity ultrasound with respect to pure water.

To evaluate the future applicability of ZnO-NH_2_ NCs in the biomedical field, the generation of ROS and the effects of NCs as ultrasound responsive nano-agent agent were tested when different tissue mimicking materials were interposed during the insonation between the US transducer and the sample well. A significant increase of cavitation is noticeable when ZnO-NH_2_ NCs are present, with respect to pure water, when phantoms and, in a larger amount, *ex vivo* tissue are interposed as the propagation medium. These measurements, together with the increased and controlled ROS production also in biological media as PBS and cell culture media (EMEM), suggest the future applicability of this technology to the *in vivo* setting.

All together these results proof the enhanced effects and controllability of ROS generation by ZnO-NH_2_ NPs assisted pulsed ultrasound, anticipating high potential in a wide range of biomedical/healthcare applications.

## Data Availability Statement

All datasets generated for this study are included in the article/Supplementary Material.

## Author Contributions

VV, AA, and LR performed most of the experiments described in the manuscript. TL designed the solution and tissue-mimicking measurements and assisted them. AT performed and evaluated critically the needle hydrophone and B-mode imaging characterization. GC designed the whole manuscript concept. VC supervised the whole work and was the recipient of the funding. All the authors contributed to the manuscript writing and corrections.

### Conflict of Interest

The authors declare that the research was conducted in the absence of any commercial or financial relationships that could be construed as a potential conflict of interest.

## References

[B1] Abdal Dayem A. Hossain M. Lee S. Kim K. Saha S. Yang G.-M.. (2017). The role of reactive oxygen species (ROS) in the biological activities of metallic nanoparticles. Int. J. Mol. Sci. 18:120. 10.3390/ijms18010120 28075405 PMC5297754

[B2] Aldosari S. Awad M. Harrington E. O. Sellke F. W. Abid M. R. (2018). Subcellular reactive oxygen species (ROS) in cardiovascular pathophysiology. Antioxidants 7:E14. 10.3390/antiox7010014 29337890 PMC5789324

[B3] Ancona A. Dumontel B. Garino N. Demarco B. Chatzitheodoridou D. Fazzini W.. (2018). Lipid-coated zinc oxide nanoparticles as innovative ROS-generators for photodynamic therapy in cancer cells. Nanomaterials 8:143. 10.3390/nano8030143 29498676 PMC5869634

[B4] Canavese G. Ancona A. Racca L. Canta M. Dumontel B. Barbaresco F.. (2018). Nanoparticle-assisted ultrasound: a special focus on sonodynamic therapy against cancer. Chem. Eng. J. 340, 155–172. 10.1016/j.cej.2018.01.060 30881202 PMC6420022

[B5] Carlson C. Hussain S. M. Schrand A. M. Braydich-Stolle L. K. Hess K. L. Jones R. L.. (2008). Unique cellular interaction of silver nanoparticles: size-dependent generation of reactive oxygen species. J. Phys. Chem. B 112, 13608–13619. 10.1021/jp712087m 18831567

[B6] Dabrowski J. M. (2017). Reactive oxygen species in photodynamic therapy: mechanisms of their generation and potentiation. Adv. Inorg. Chem. 70, 343–394. 10.1016/bs.adioch.2017.03.002

[B7] de Sá Junior P. L. Câmara D. A. D. Porcacchia A. S. Fonseca P. M. M. Jorge S. D. Araldi R. P.. (2017). The roles of ROS in cancer heterogeneity and therapy. Oxid. Med. Cell. Longev. 2017:2467940. 10.1155/2017/2467940 29123614 PMC5662836

[B8] Dolmans D. Fukumura D. Jain R. (2003). Photodynamic therapy for cancer. Nat. Rev. Cancer. 3:8. 10.1038/nrc1071 12724736

[B9] Dos Santos A. F. De Almeida D. R. Q. Terra L. F. Baptista M. S. Labriola L. (2019). Photodynamic therapy in cancer treatment - An update review. J. Cancer Metastasis Treat. 5:25 10.20517/2394-4722.2018.83

[B10] Dougherty T. J. Gomer C. J. Henderson B. W. Jori G. Kessel D. Korbelik M.. (1998). Photodynamic therapy. J. Natl. Cancer Inst. 90:17. 10.1093/jnci/90.12.889 9637138 PMC4592754

[B11] Dumontel B. Canta M. Engelke H. Chiodoni A. Racca L. Ancona A.. (2017). Enhanced biostability and cellular uptake of zinc oxide nanocrystals shielded with a phospholipid bilayer. J. Mater. Chem. B 5, 8799–8813. 10.1039/C7TB02229H 29456858 PMC5779080

[B12] Firdous S. (2018). Development and imaging of zinc oxide nanorods as a photosensitizer for the diagnosis and treatment of cancer using lasers. Laser Phys. Lett. 15:095604 10.1088/1612-202X/aad28c

[B13] Fu P. P. Xia Q. Hwang H.-M. Ray P. C. Yu H. (2014). Mechanisms of nanotoxicity: generation of reactive oxygen species. J. Food Drug Anal. 22, 64–75. 10.1016/j.jfda.2014.01.005 24673904 PMC9359151

[B14] Garino N. Limongi T. Dumontel B. Canta M. Racca L. Laurenti M.. (2019a). A microwave-assisted synthesis of zinc oxide nanocrystals finely tuned for biological applications. Nanomaterials 9:212. 10.3390/nano9020212 30736299 PMC6410313

[B15] Garino N. Sanvitale P. Dumontel B. Laurenti M. Colilla M. Izquierdo-Barba I.. (2019b). Zinc oxide nanocrystals as a nanoantibiotic and osteoinductive agent. RSC Adv. 9, 11312–11321. 10.1039/C8RA10236H 31024686 PMC6478122

[B16] Gonzalez L. Lison D. Kirsch-Volders M. (2008). Genotoxicity of engineered nanomaterials: a critical review. Nanotoxicology 2, 252–273. 10.1080/17435390802464986

[B17] Hu Z. Li J. Li C. Zhao S. Li N. Wang Y. (2013). Folic acid-conjugated graphene–zno nanohybrid for targeting photodynamic therapy under visible light irradiation. J. Mater. Chem. B 1:5003 10.1039/c3tb20849d 32261090

[B18] Kim Y.-M. Kim S.-J. Tatsunami R. Yamamura H. Fukai T. Ushio-Fukai M. (2017). ROS-induced ROS release orchestrated by Nox4, Nox2, and mitochondria in VEGF signaling and angiogenesis. Am. J. Physiol. Cell Physiol. 312, C749–C764. 10.1152/ajpcell.00346.2016 28424170 PMC5494593

[B19] Lau A. T. Y. Wang Y. Chiu J.-F. (2008). Reactive oxygen species: current knowledge and applications in cancer research and therapeutic. J. Cell. Biochem. 104, 657–667. 10.1002/jcb.21655 18172854

[B20] Lops C. Ancona A. Di Cesare K. Dumontel B. Garino N. Canavese G.. (2019). Sonophotocatalytic degradation mechanisms of rhodamine B dye via radicals generation by micro- and nano-particles of ZnO. Appl. Catal. B Environ. 243, 629–640. 10.1016/j.apcatb.2018.10.078 30886458 PMC6420045

[B21] McHale A. P. Callan J. F. Nomikou N. Fowley C. Callan B. (2016). Sonodynamic therapy: concept, mechanism and application to cancer treatment, in Therapeutic Ultrasound, Vol. 880, eds Escoffre J.-M. Bouakaz A. (Cham: Springer International Publishing), 429–450. 10.1007/978-3-319-22536-4_22 26486350

[B22] Nogueira V. Hay N. (2013). Molecular pathways: reactive oxygen species homeostasis in cancer cells and implications for cancer therapy. Clin. Cancer Res. 19, 4309–4314. 10.1158/1078-0432.CCR-12-1424 23719265 PMC3933310

[B23] Osumi K. Matsuda S. Fujimura N. Matsubara K. Kitago M. Itano O. (2017). Acceleration of wound healing by ultrasound activation of TiO _2_ in *Escherichia coli*-infected wounds in mice: wound healing with sonicated TiO_2_. J. Biomed. Mater. Res. B Appl. Biomater. 105, 2344–2351. 10.1002/jbm.b.33774 27507677

[B24] Pelicano H. Carney D. Huang P. (2004). ROS stress in cancer cells and therapeutic implications. Drug Resist. Updat. 7, 97–110. 10.1016/j.drup.2004.01.004 15158766

[B25] Racca L. Canta M. Dumontel B. Ancona A. Limongi T. Garino N. (2018). Zinc oxide nanostructures in biomedicine, in Smart Nanoparticles for Biomedicine, ed Ciofani G. (Amsterdam: Elsevier Inc), 171–187. 10.1016/B978-0-12-814156-4.00012-4

[B26] Shafique E. Choy W. C. Liu Y. Feng J. Cordeiro B. Lyra A.. (2013). Oxidative stress improves coronary endothelial function through activation of the pro-survival kinase AMPK. Aging 5, 515–530. 10.18632/aging.100569 24018842 PMC3765580

[B27] Tong L. Chuang C.-C. Wu S. Zuo L. (2015). Reactive oxygen species in redox cancer therapy. Cancer Lett. 367, 18–25. 10.1016/j.canlet.2015.07.008 26187782

[B28] Troia A. Cuccaro R. Schiavi A. (2017). Independent tuning of acoustic and mechanical properties of phantoms for biomedical applications of ultrasound. Biomed. Phys. Eng. Express. 3:025011 10.1088/2057-1976/aa5ed0

[B29] Urao N. Inomata H. Razvi M. Kim H. W. Wary K. McKinney R.. (2008). Role of Nox2-based NADPH oxidase in bone marrow and progenitor cell function involved in neovascularization induced by hindlimb ischemia. Circ. Res. 103, 212–220. 10.1161/CIRCRESAHA.108.176230 18583711 PMC2711765

[B30] van Straten D. Mashayekhi V. de Bruijn H. Oliveira S. Robinson D. (2017). Oncologic photodynamic therapy: basic principles, current clinical status and future directions. Cancers 9:19. 10.3390/cancers9020019 28218708 PMC5332942

[B31] Yasuda J. Miyashita T. Taguchi K. Yoshizawa S. Umemura S. (2015). Quantitative assessment of reactive oxygen sonochemically generated by cavitation bubbles. Jpn. J. Appl. Phys. 54 (S1):07HF21 10.7567/JJAP.54.07HF21

[B32] Zhang H. Chen B. Jiang H. Wang C. Wang H. Wang X. (2011). A strategy for ZnO nanorod mediated multi-mode cancer treatment. Biomaterials 32, 1906–1914. 10.1016/j.biomaterials.2010.11.027 21145104

