# Supplementary material for: The Synergistic Effect of Nanocrystals Combined With Ultrasound in the Generation of Reactive Oxygen Species for Biomedical Applications
Source: Front Bioeng Biotechnol. 2019 Nov 26;7:374. doi: 10.3389/fbioe.2019.00374 (PMC6988813; doi:10.3389/fbioe.2019.00374)

**SUPPORTING INFORMATION**

**The synergistic effect of nanocrystals combined with ultrasound in the generation of reactive oxygen species for biomedical applications**

**Veronica Vighetto^1+^, Andrea Ancona^1+^, Luisa Racca^1^, Tania Limongi^1^, Adriano Troia^2^, Giancarlo Canavese^1^, Valentina Cauda^1^***

^1^Department of Applied Science and Technology, Politecnico di Torino, C.so Duca degli Abruzzi 24, 10129 Turin, Italy

^2^Ultrasounds & Chemistry Lab, Advanced Metrology for Quality of Life, Istituto Nazionale di Ricerca Metrologica (I.N.Ri.M.) Strada delle Cacce 91, 10135 Turin, Italy

Temperature in the well was monitored during 20 min of US irradiation with 50% DC, 1 MHz frequency, 3 W/cm^2^ in water using the LipoZero transducer (these correspond to the maximum time, DC and power used in our work). We observe an initial increase of maximum 5 degree, than the temperature remains constant during the ultrasound irradiation. To measure the temperature Multilogger Thermometer 502A1 (TERSID S.r.l.) was used. Measurements were conducted in duplicate. Result are reported here below:


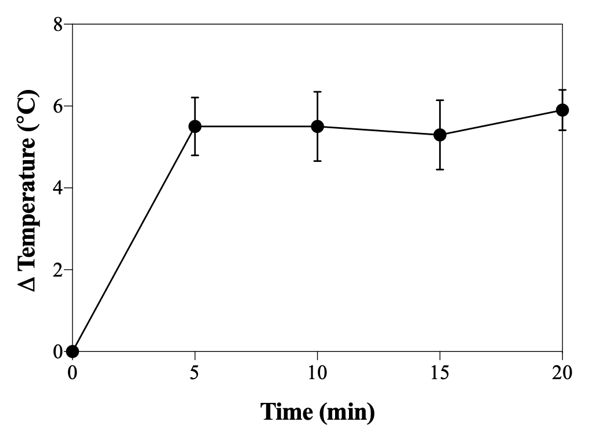


ROS generation was evaluated in PBS solution and in complete cell culture medium. Cell culture medium used for these measurements was the Minimum Essential Medium Eagle (SIGMA) completed with 10% of Fetal Bovine Serum (FBS, SIGMA) and 1% of Penicillin-Streptomycin. The ultrasound irradiation was performed at 10% DC, 0.9 W/cm^2^, 10 min. The results confirm the ability of our ZnO-NH_2_ nanocrystals to enhance inertial cavitation and consequently ROS production also in biological media. ROS evaluation were performed as reported in section ‘Materials and Methods’ of this work.


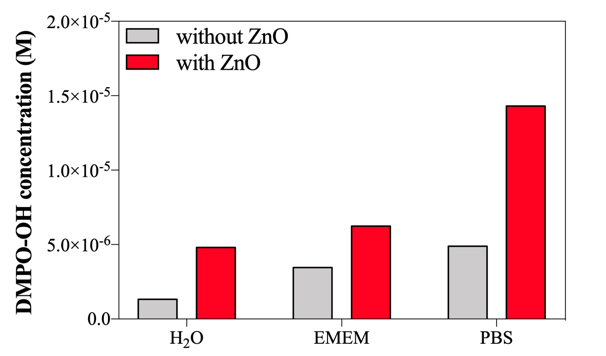

Supplement: Supplementary file 1 [file Table_1.DOCX]
